# Supplementary material for: Modular, focused data science education improves biomedical learners’ abilities: A study of the Data and Analytics for Research Training (DART) program
Source: PLoS Comput Biol. 2025 Jul 17;21(7):e1013249. doi: 10.1371/journal.pcbi.1013249 (PMC12286386; doi:10.1371/journal.pcbi.1013249)
Supplement: Attachment — Submitted filename: Reviewer Rec - NT Msweli.pdf [file pcbi.1013249.s001.pdf]

Recommendation and Comments for Manuscript Number PCOMPBIOL-D-24-02043

- *What are the main claims of the paper and how significant are they for the discipline?*

  The paper makes two primary claims.

  - First, it asserts that modular, tailored data science training programs can significantly enhance learners' self-assessed technical skills in data science within biomedical research.
  - Second, it highlights a moderate impact on participants' attitudes toward open science principles, with limitations due to pre-existing high agreement among participants.

  These findings are significant for the discipline as they address gaps in specialized, scalable, and adaptable training for biomedical researchers, emphasizing the importance of tailored educational pathways in improving technical competency and promoting rigorous, reproducible research practices. Moreover, the study provides a replicable framework for evaluating educational interventions, contributing to ongoing efforts to refine data science education in the biomedical domain.

- *Are these claims novel? If not, which published articles weaken the claims of originality of this one?*

The claims are partially novel. While the development of tailored, modular, and scalable training programs is significant, similar efforts have been described in prior research.

- Chen (2022) described a pedagogical approach tailored to biomedical data science education, including modular content and assessments based on learner profiles. This parallels the tailored pathways emphasized in the DART program.
- Auer et al. (2021) proposed a large-scale training program for reproducibility in life sciences, also using modular content and focusing on researcher needs. This overlap weakens the novelty of focusing on reproducibility and modular learning.
- Federer et al. (2016) outlined the data literacy needs of biomedical researchers and proposed training programs designed around those needs. This undermines the claim of addressing unmet educational needs specific to biomedical researchers.

- *Are the claims properly placed in the context of the previous literature? Have the authors treated the literature fairly?*

The authors of the paper have partially placed their claims in the context of the previous literature, but there are areas where the treatment of prior work could be improved to provide a more comprehensive and balanced view.

- The authors reference similar efforts, but they do not sufficiently explore how the DART program compares or builds upon these initiatives.
- The authors briefly mention the scalability issues of workshop-based training but do not critically analyze whether their asynchronous approach faces limitations, such as reduced learner engagement, as highlighted in related studies.
- The literature on limitations of self-reported measures in educational studies and challenges in sustaining engagement in asynchronous programs is not discussed, leaving a gap in critically contextualizing their findings.

While the authors have treated the literature fairly in terms of acknowledging key prior works, they fall short of fully situating their claims in the broader context. A deeper engagement with

closely related studies, critical analysis of overlaps, and acknowledgment of potential limitations in their approach would have strengthened their argument and enhanced the credibility of their claims. As it stands, their treatment of the literature leans slightly toward emphasizing novelty without fully addressing the precedents.

- *Do the data and analyses fully support the claims? If not, what other evidence is required?*

The data and analyses presented in the paper largely support the claims. For instance:

- The data show statistically significant improvements in self-reported data science skills across two waves of participants. Effect sizes are large, especially in Wave 1 (Cohen's d = 1.47), and are consistent across analyses. This supports the claim that the DART program improves learners' technical skills.

- The study could make future recommendations on identified limitations. For instance; the reliance on self-reported measures for skill improvement is a significant limitation. Self-assessments can be biased by participants' confidence or perception rather than actual skill acquisition. Future studies could look at objective measures of learning, such as task-based assessments or evaluations by external experts, which would strengthen the claims.

- *Would additional work improve the paper? How much better would the paper be if this work were performed and how difficult would it be to do this work?*

Additional work could improve the paper by addressing key limitations and providing a more robust foundation for its claims.

- Incorporating objective measures of skill acquisition, such as performance-based assessments or third-party evaluations, would validate self-reported improvements and strengthen the findings.
- Introducing a randomized control group or a quasi-experimental design would clarify the causal relationship between the DART program and the observed outcomes, making the claims more convincing.
- Conducting follow-up studies to assess the long-term retention of skills and their application in research practice would elevate the paper's impact and relevance.

While implementing these measures involves moderate difficulty, requiring additional time, resources, and participant recruitment, the improvements in credibility, rigor, and practical applicability of the study's findings would make the paper substantially better and more influential in the field.

- *PLOS Computational Biology encourages authors to publish detailed protocols and algorithms as supporting information online. Do any particular methods used in the manuscript warrant such treatment? If a protocol is already provided, for example for a randomized controlled trial, are there any important deviations from it? If so, have the authors explained adequately why the deviations occurred?*

The manuscript introduces a pre-registered study design and describes the Data and Analytics for Research Training (DART) program, which incorporates tailored, modular educational pathways and uses hierarchical clustering to assign learners to relevant pathways. These methodologies, particularly the clustering approach and the modular program design, warrant publication as detailed protocols or algorithms in the supporting information to enhance reproducibility and enable adaptation by other researchers. While the manuscript references

pre-registration and adherence to planned hypotheses, it also notes changes between Wave 1 and Wave 2, such as modifications to the engagement metric and learning management system. These deviations are adequately explained as responses to feedback and limitations observed in Wave 1, ensuring the integrity of the research process.

- *Are original data deposited in appropriate repositories and accession/version numbers provided for genes, proteins, mutants, diseases, etc.?*

The authors have deposited the original data and analysis scripts in appropriate repositories. They provide a GitHub repository link for the Data and Analytics for Research Training (DART) analysis, along with a Zenodo DOI for versioned access to the repository.

- *Has the author-generated code that underpins the findings been made publicly available?*

  The authors have made the code that underpins their findings publicly available. They have deposited the code in a GitHub repository and assigned it a Zenodo DOI for proper citation and versioning.

- *Are details of the methodology sufficient to allow the experiments to be reproduced?*

  The manuscript provides sufficient details for reproducing the experiments to a reasonable extent, including descriptions of the DART program's modular structure

- *Is any software created by the authors freely available?*

  The authors have made the software they used for their study freely available. They developed and shared educational modules and analytical tools through a public GitHub repository, which is also linked to a Zenodo DOI for versioning and citation.

- *Is the manuscript well organized and written clearly enough to be accessible to non-specialists?*

  The manuscript is generally well-organized and written clearly, making it accessible to readers with a broad range of expertise. The authors provide a logical structure, starting with an overview of the problem, detailing the DART program’s design and implementation, and presenting results supported by clear figures and statistical analyses. The language is concise and avoids excessive technical jargon, which enhances its accessibility to non-specialists.

  Some sections, such as the clustering algorithm and statistical modeling details, may benefit from further simplification or additional explanation to ensure full clarity for readers unfamiliar with these methods.

- *Does the paper use standardized scientific nomenclature and abbreviations? If not, are these explained at the first usage?*

  The paper generally adheres to standardized scientific nomenclature and explains abbreviations at their first usage
