# Supplementary material for: Modular, focused data science education improves biomedical learners’ abilities: A study of the Data and Analytics for Research Training (DART) program
Source: PLoS Comput Biol. 2025 Jul 17;21(7):e1013249. doi: 10.1371/journal.pcbi.1013249 (PMC12286386; doi:10.1371/journal.pcbi.1013249)
Supplement: Attachment — Submitted filename: Response to reviewers.docx [file pcbi.1013249.s003.docx]

We are very grateful to both reviewers for their careful reading of the manuscript and their thoughtful feedback. Their reviews were clear, insightful, and productive, and we believe the manuscript is much stronger as a result of their contributions. In response to their feedback, we substantially expanded our discussion and clarified the text in several places:

- Reviewer 2 (R2) pointed out the high level of attrition in our study, and the resulting risk of bias in our results. This was also highlighted in the letter from the editor. We added discussion drawing the reader’s attention to this important limitation of our work, to better contextualize our findings. We also ran additional analyses more closely examining, when possible, differences between learners who completed the program vs. those who did not (full analysis available as a report in our analysis repository: <https://github.com/arcus/DART_analysis/blob/main/reports/notebooks/attrition_analysis.md>) and added a couple key insights from that analysis into our discussion of attrition in our study. We also added a little new content about the issue of attrition in studies of online education (including three new citations: 24-26), as it is an important challenge in the field.
- Reviewer 1 (R1) asked that we spend more time in our review of the previous literature clearly defining DART relative to other educational programs, more clearly explaining how it compares and where it builds. Our program was inspired by several other existing programs, as mentioned in the Introduction, but we see our contribution as an important departure from that existing work because of our focus on exclusively asynchronous instruction. We added new language to the introduction to make that distinction clearer.
- R1 asked that we more thoroughly address the shortcomings of asynchronous education instead of focusing exclusively on its strengths. We added new content discussing this in both the Introduction and Discussion sections (referencing six new citations: 17, 18, 27-30), better contextualizing both our decisions in designing DART and our results and potential future directions.
- R1 noted that we did not sufficiently address the limitations of relying on only self-report measures (e.g. we may have been measuring changes in participants’ confidence or self-efficacy rather than actual growth in skill). This was echoed in the letter from the editor. We added new language to the Discussion section addressing this (including two new citations: 21,22), drawing the reader’s attention to this alternate interpretation of our findings and also providing additional justification for our approach and highlighting directions for future work.
- R1 recommended we consider several additions to the study to strengthen the findings, including objective measures of learning (e.g. task-based assessments or evaluations by external experts) to validate self-reports, long term follow-up to assess retention of skills and their application in research practice, and a randomized control group or other improved research design that would allow us to clarify the causal role of DART (if any) on the observed outcomes. We wholeheartedly agree that each of these suggestions would substantially strengthen our work. We lack the staffing and funding to run another wave of the DART study at this time, however, so we are unable to implement these suggestions for the current manuscript. We did add language discussing these as valuable future directions, and emphasizing that all of our materials (including the educational content itself) are freely available should another team want to act on those suggestions.
- R1 also suggested that we review our descriptions of the clustering algorithm and statistical modeling details to see if we could simplify and clarify those sections for readers unfamiliar with those methods. We did make adjustments to both descriptions, and we think the new text reads much clearer.
