# Supplementary material for: Modular, focused data science education improves biomedical learners’ abilities: A study of the Data and Analytics for Research Training (DART) program
Source: PLoS Comput Biol. 2025 Jul 17;21(7):e1013249. doi: 10.1371/journal.pcbi.1013249 (PMC12286386; doi:10.1371/journal.pcbi.1013249)
Supplement: Attachment — Submitted filename: Response_to_Reviewers_auresp_2.docx [file pcbi.1013249.s004.docx]

We are very grateful to both reviewers for their review and to the editor for their additional commentary supporting the reviewers’ feedback. Reviewer 1 provided additional feedback that we have addressed, and we describe our changes to the manuscript here:

- Reviewer 1 rightly points out, “*While the study title is proper and descriptive, authors should consider shortening the title.*” We agree that the original title, “Modular data science education, tightly scoped for biomedicine, improves learners’ technical abilities: A study of the Data and Analytics for Research Training (DART) Program”, is indeed wordy. We have shortened it to “Modular, focused data science education improves biomedical learners’ abilities: A study of the Data and Analytics for Research Training (DART) Program.” We believe this captures the intent to highlight the program’s specific characteristics while being more readable.
- Reviewer 1 suggests: “*The authors mentions ‘There are many examples of high-quality data science educational options.’ it would be nice if they can provide these examples.*” We would like to offer some context for our cited language as a rebuttal. We follow the quoted sentence in our manuscript by offering three categories of educational categories: “data science degree programs,” “Other options, such as certificate programs and free or inexpensive online course platforms,” and “recent projects.” In the final two of the three categories, which we anticipate being less familiar to academic readers due to their existence outside of traditional degree-granting structures, we do offer named examples and offer additional unnamed citations. We do not, however, as Reviewer 1 points out, provide examples in the first category, that of formal degree programs. We feel that it is unnecessary and may constitute bias to highlight specific examples of “data science degree programs.” There are a staggering number of potential worthy candidates to choose from across thousands of degree-granting institutions in the United States and abroad. We imagine that most of our readers are affiliated with or know of institutions which offer data science degrees, and we do not wish to endorse any particular program.
- Reviewer 1 remarks, “*The discussion section needs revision. Currently, it places too much emphasis on limitations, which overshadows the core purpose of this section — that is, to interpret, contextualize, and critically analyze the findings of the study.”* We are grateful for this reminder and suggestion. We revised the discussion section, removing much of the content related solely to future directions and refocusing on contextualizing and interpreting the results of the present study.
